# Establishment of an Intradermal Ear Injection Model of IL-17A and IL-36γ as a Tool to Investigate the Psoriatic Cytokine Network

**DOI:** 10.3390/life11080846

**Published:** 2021-08-19

**Authors:** David Kluwig, Sebastian Huth, Ali T. Abdallah, Carolina M. Pfaff, Katharina Fietkau, Laura Huth, Yvonne Marquardt, Jens M. Baron, Bernhard Lüscher

**Affiliations:** 1 Institute of Biochemistry and Molecular Biology, RWTH Aachen University, 52074 Aachen, Germany; carolina.pfaff@rwth-aachen.de (C.M.P.); bluescher@ukaachen.de (B.L.); 2 Department of Dermatology and Allergology, Faculty of Medicine, RWTH Aachen University, 52074 Aachen, Germany; shuth@ukaachen.de (S.H.); kfietkau@ukaachen.de (K.F.); lhuth@ukaachen.de (L.H.); ymarquardt@ukaachen.de (Y.M.); jbaron@ukaachen.de (J.M.B.); 3 Interdisciplinary Center for Clinical Research (IZKF), Faculty of Medicine, RWTH Aachen University, 52074 Aachen, Germany; bioinformatics.ali.abdallah@gmail.com

**Keywords:** psoriasis, mouse model, IL-17A, IL-36γ, ear injection

## Abstract

Psoriasis is a chronic skin disease affecting 2–3% of the global population. The proinflammatory IL-17A is a key cytokine in psoriasis. Accumulating evidence has revealed that IL-36γ plays also a pathogenic role. To understand more precisely the role of the IL-17A–IL-36γ cytokine network in skin pathology, we used an ear injection model. We injected IL-17A or IL-36γ alone and in combination into the ear pinnae of mice. This resulted in a significant increase in ear thickness measured over time. Histological evaluation of IL-17A + IL-36γ-treated skin showed a strong acanthosis, hyperparakeratosis and infiltration of neutrophils. The same histological features were found in mice after injection of IL-36γ alone, but to a lesser extent. IL-17A alone was not able to induce psoriasis-like changes. Genes encoding proteins of the S100 family, antimicrobial peptides and chemo-attractants for neutrophils were upregulated in the IL-17A + IL-36γ group. A much weaker expression was seen after the injection of each cytokine alone. These results strengthen the hypothesis that IL-17A and IL-36γ drive psoriatic inflammation via a synergistic interaction. Our established intradermal ear injection model can be utilized in the future to monitor effects of various inhibitors of this cytokine network.

## 1. Introduction

Psoriasis is a chronic skin disease caused by the excessive secretion of inflammatory cytokines affecting 2–3% of the global population [1]. Psoriatic skin lesions are associated with increased infiltration of immune cells, which includes different subpopulations of T cells and neutrophils. These cells affect the cytokine milieu and thus the behavior of keratinocytes, including their altered differentiation. It was reported that type 1 helper T (TH1) cells, whose development is stimulated by IL-12, are increased in psoriatic lesions, and associated proinflammatory cytokines, including tumor necrosis factor (TNF) α and interferon (IFN) γ, are elevated [2,3,4]. In addition, T helper 17 (TH17) cells are critical for the psoriatic phenotype [5,6]. This is also undermined by the strong psoriasis-like skin phenotype upon transgenic expression of IL-17A, a key cytokine produced by TH1 cells, under the control of the keratin-14 promoter in mice [7]. In addition to IL-17, IL-23 is also involved in psoriasis. IL-23 is produced mainly by dendritic cells and macrophages and activates TH17 cells, thereby promoting IL-17 production. It has been suggested that the IL-23 effects are dependent on IL-17A [8,9,10], which is also consistent with the findings that neutralizing anti-IL-17 antibodies, including Secukinumab and Ixekizumab, are approved as first-line treatment of moderate-to-severe plaque psoriasis [11,12]. These drugs showed effectiveness in all the various subtypes of psoriasis and in various TH17-related conditions [13,14]. Blocking the p19 subunit of IL-23 with selective antibodies such as Guselkumab and Risankizumab inhibits the intracellular and downstream signaling of IL-23 and is highly effective in the treatment of psoriasis [15,16]. In addition to the IL-23–IL-17 axis [17], evidence is accumulating that IL-36 cytokines are essential in psoriasis [18,19]. The three members, IL-36α, β, and γ, encoded by *IL36A*, *B* and *C* in humans and *Il1F6*, *8* and *9* in mice, belong to the IL-1 cytokine family and possess multiple roles in host immunity and several inflammatory diseases [20,21]. Among the IL-36 members, IL-36γ plays a dominant role in the pathomechanism of psoriasis [18,19]. So far, using keratinocyte monolayers and an in vitro 3D psoriasis model, we observed that IL-17A induces IL-36 cytokines and that a feedback loop between IL-17 and IL-36 cytokines provokes and maintains psoriasis pathology [22]. The role of IL-36 was further demonstrated by using IL-36R antagonistic antibodies in a mouse model of psoriasis, which is induced by the TLR7 agonist imiquimod [23], and which ameliorates the phenotype [24]. It is highly expressed in psoriasis lesions, produced by epidermal keratinocytes, and to a lesser extent by dermal fibroblasts and endothelial cells. Blood serum levels of IL-36 are associated with disease activity [25]. It was reported that treatment with the anti-TNF-α drug etanercept decreases IL-36γ serum levels [25]. Additionally, topical treatment with calcipotriol suppresses IL-36γ expression in psoriasis plaques [26]. Furthermore, small molecules have been developed and tested in preclinical models of psoriasis that inhibit neutrophil proteases, which are responsible for the activation of IL-36 [27]. A phase 1 study with the IL-36 receptor inhibitor BI655130 showed beneficial effects in generalized pustular psoriasis after application of one intravenous dose [28]. Generalized pustular psoriasis is a potentially life-threatening distinct form of psoriasis. Importantly, it was noted that a homozygous mutation in the gene encoding the IL-36 receptor antagonist reduces its expression and function, promoting unregulated secretion of inflammatory cytokines associated with generalized pustular psoriasis [29]. Il-36 cytokines also play a role in palmoplantar pustulosis (PPP), which is much more common and difficult to treat. In psoriatic arthritis (PsA) high levels of IL-36 cytokines were found in synovium and appear to play a role in synovial inflammation [30]. To further evaluate the role of the IL-17A–IL-36γ cytokine axis for the pathomechanism of psoriasis, we established an ear injection model. We found that IL-17A and IL-36γ cooperate in promoting a psoriasis-like phenotype and infiltration of neutrophils. Moreover, a gene expression program is activated that is comparable to alterations seen in psoriasis.

## 2. Materials and Methods

### 2.1. Animals

Eight-week-old female C57BL/6 mice were used. They were purchased from Janvier (Le Genest-Saint-Isle, France). All animal procedures and experiments were conducted in accordance with the German federal law regarding the protection of animals. Protocols were approved by the administration of the “Landesamt für Umwelt, Natur und Verbraucherschutz” (LANUV, Recklinghausen, Germany). Each group consisted of 5 animals.

### 2.2. Injection of Cytokines and Antibodies

The following recombinant murine cytokines, diluted in PBS, were used: IL-17A (PeproTech 210-17, East Windsor, NJ, USA) and IL-36γ (BioLegend 552806, San Diego, CA, USA). The cytokines IL-17A and IL-36γ were carefully injected intradermally, either separately or combined, into the ear pinnae (1 µg of cytokine per ear dissolved in PBS; volume 5 µL) by using a Hamilton Syringe (33 gauge). The control group received injections with PBS. Accordingly, four groups (PBS, IL-17A, IL-36γ, IL-17A + IL-36γ) were included in the study.

### 2.3. Ear Thickness Measurement

Ear thickness measurement was performed daily with a pocket thickness gauge (Hitec Messtechnik GmbH, Magstadt, Germany). The measuring tool was positioned at the ear pinnae of the mice.

### 2.4. Histology and Immunohistochemistry

After four days of consecutive treatment, the mice were euthanized, and both ears were collected. One part of the ear was fixed in formalin and embedded in paraffin, followed by haematoxylin and eosin (H&E) staining. Moreover, cryosections of the ears were stained for neutrophils with a Ly-6G antibody (553125 BD, Franklin Lakes, NJ, USA).

### 2.5. Light Microscopy

Examination and photographic documentation of the H&E-stained paraffin-embedded sections of formalin-fixed ear tissue and the cryosections were performed using a DMIL microscope (Leica, Wetzlar, Germany).

### 2.6. RNA Preparation, Reverse Transcription, and Quantitative RT-PCR

One part of the ear was harvested for RNA isolation. Quantitative RT-PCR experiments were performed directly afterwards. According to the manufacturer’s instructions, the Nucleo Spin RNA kit (Macherey-Nagel, Düren, Germany) was used for total RNA extraction. Residual genomic DNA was removed by rDNase (RNase-free) digestion. The tissues of the mouse ears were mechanically disrupted and homogenized by using a tissue lyser (Qiagen, Hilden, Germany). A total of 1 µg of RNA was reverse transcribed into cDNA by utilizing High Capacity RNA-to-cDNA Master Mix (Applied Biosystems, Foster City, CA, USA). TaqMan experiments were carried out on an ABI PRISM 7300 sequence detection system (Applied Biosystems, Foster City, CA, USA) using Assay-on-Demand gene expression products (Applied Biosystems, Foster City, CA, USA) for Defb4 (Mm00731768_m1) and S100a8 (Mm00496696_g1). An Assay-on-Demand product for Hprt mRNA (Mm00446968) was used as an internal reference to normalize the target transcripts.

### 2.7. RNA-Sequencing

We have randomly selected 3 out of 5 initially generated samples and subjected them to an RNA-Sequencing experiment, consisting of library preparation, sequencing, and subsequent analysis.

#### 2.7.1. Library Preparation

Quality checks of RNA samples were performed with the TapeStation 4200 using the Agilent RNA ScreenTape Assay kit. Quantification was performed using the Quantus RNA System (Promega, Madison, WI, USA). cDNA libraries were produced employing the TrueSeq Stranded Total RNA Library Preparation Kit with Ribo-Zero Gold Kit according to the manufacturer’s instructions (Illumina Inc., San Diego, CA, USA), using 500 ng input RNA or 10 µL for the immunoprecipitated samples, respectively. The cDNA libraries’ quality and quantity were assessed using the 4200 TapeStation (D1000 screen tape assay) and the Quantus dsDNA system, respectively.

#### 2.7.2. Sequencing

The libraries were run on an Illumina NextSeq 500 platform using the High Output 150 cycles kit (paired-end reads, 76 cycles for read 1, 76 cycles for read 2, and 6 cycles for index 1). The run includes the primary analysis workflow, mainly consisting of template generation, imaging, and base calling using RTA 2.0 onsite. Then, the run resulted in an average sample size of 88.45 million reads and a sample size standard deviation of 21.23 million reads. The samples were sequenced on a NextSeq 500 sequencing system with the following run parameters: 76 cycles for read 1, 76 cycles for read 2, and 6 cycles for index 1. The cDNA libraries were generated using a TruSeq Stranded Total RNA Sample Preparation Kit (Illumina Inc., San Diego, CA, USA).

#### 2.7.3. RNA-Seq Analysis

The RNA-Seq primary analysis was performed with an in-house pipeline embedded in the QuickNGS workflow management system [31]: the generation of the fastq files and adapter removal was completed using the Illumina demultiplexing and conversion software bcl2fastq (https://support.illumina.com/sequencing/sequencing_software/bcl2fastq-conversion-software.html (accessed on 14 January 2020)). Quality control checks of the RNA-Seq data were performed with fastqc, available online (http://www.bioinformatics.babraham.ac.uk/projects/fastqc (accessed on 14 January 2020)). Subsequently, the reads were aligned to the mm10 reference genome sequence using STAR (v2.5.2b) with default parameters [32]. In the quantification step, reads were counted with featureCounts from the subread package v1.5.1 available online (http://subread.sourceforge.net (accessed on 14 January 2020)) [33].

#### 2.7.4. Differential Expression Analysis

Differential expression analysis was performed in R using the DESeq2 package (v1.30.0) [34]: first, the size factors of each sample were estimated using the function estimateSizeFactors with default parameters, then the dispersions of the counts were estimated by setting the minimal dispersion to 10e-6 and setting the fit type parameter to “local”. Finally, the likelihood ratio test (chi-squared test) for GLMs (negative binomial likelihood ratio test) was used to generate the differentially expressed genes (log2 fold change > 1 and corrected *p*-value < 0.05). *p*-values were corrected for multiple testing with the p.adjust function from the R package stats (v 4.0.3) using the Benjamini Hochberg correction.

#### 2.7.5. Gene Set Enrichment Analysis

Gene Set Enrichment Analysis was performed with the piano package (v 2.6.0) using the “runGSA” method [35]. For statistical gene set analysis (GSA), we used the Fisher, Stouffer, and Reporter methods successively. To assess the gene set significance, we have used the null distribution. To correct for multiple tests, we have used the Benjamini Hochberg method. Gene sets enriched in at least two of the three used GSA methods and positively enriched either by Stouffer or by Reporter methods (the Fisher method is non-directional), which were used to select the 19 gene sets. A gene set was considered enriched if it has an adjusted *p*-value of at most 0.05.

#### 2.7.6. Heatmap of the Top Differentially Expressed Genes in the Selected GO Terms

We have selected from each selected gene set all upregulated genes with high significance in the combined injection compared to PBS injection, with genes with a fold change ≥2 and a *p*-value < 0.01 considered as highly significant. Gene counts were normalized gene-wise. Each measured expression value of a gene was divided by the sum of this gene’s expression values across all samples and upscaled by a scaling factor of 10^6^. Afterward, each normalized value was centered around the average expression of this gene across all samples. Finally, the counts were rescaled by the thousandfold of the maximal normalized count. Based on this, we constructed the heatmap matrix of the selected GO-terms and generated the heatmap with the R-package pheatmap (v1.0.12).

#### 2.7.7. Heatmap of the Top 50 Variable Genes

The heatmap of the top 50 variable genes was generated using the same normalization, scaling, and centering procedure described for the heatmap of GO-terms. The top 50 genes were selected based on their variability across all samples. We have used the variance as a measure of variability.

### 2.8. Statistical Analysis of the Ear Thickness and Epidermal Thickness Measurement

Data are given as arithmetical means ± standard deviation (SD). Mann–Whitney U test and Bonferroni multiple comparisons tests were performed with GraphPad PRISM version 7 (La Jolla, CA, USA). Values of * *p* < 0.05, ** *p* < 0.01 and *** *p* < 0.001 were considered significant and are indicated in the figures.

## 3. Results

**Figure 1:**
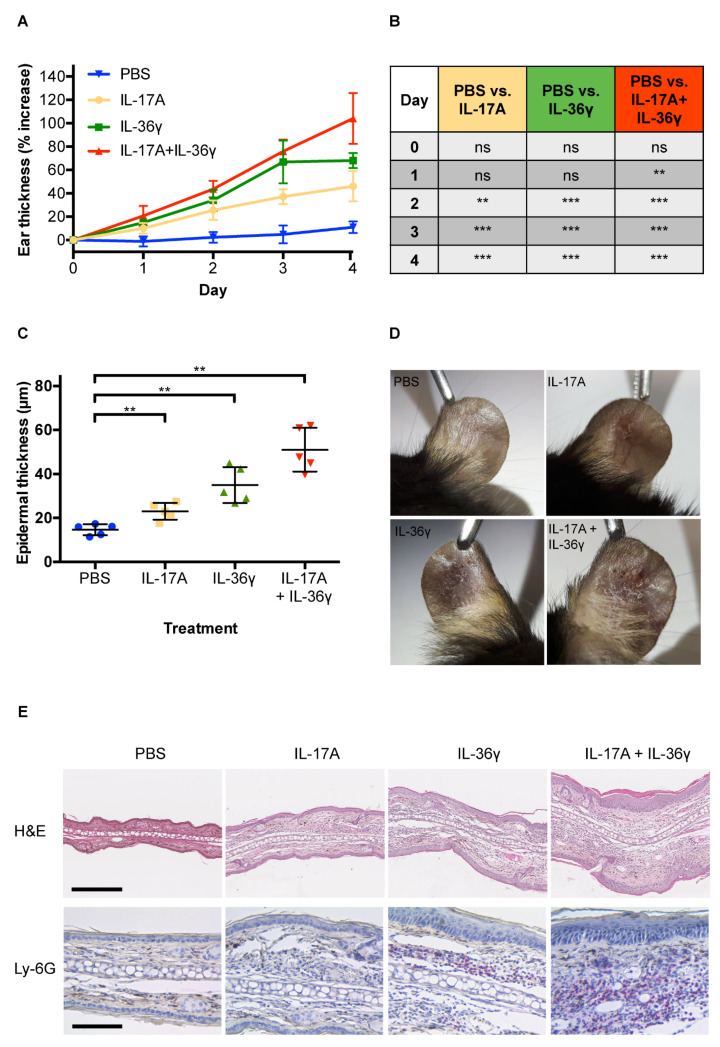
Intradermal delivery of IL-17A and IL-36γ resulted in a significant increase of ear thickness measured, lead to psoriasis-like changes and recruitment of neutrophils. (**A**) Ear thickness was measured daily in C57BL/6J mice treated either with PBS, IL-17A, IL-36γ or the combination of both cytokines. Data show an increase of ear thickness expressed as a percentage increase from the baseline values on day 0. (**B**) A separate table shows significant differences in the increase in ear thickness in each group compared to the PBS-treated group. ** *p* < 0.01, *** *p* < 0.001. (**C**) Epidermal thickness was measured in two representative H&E stained tissue sections from each mouse. Measurement was carried out at four positions per tissue section. ** *p* < 0.01. (**D**) Representative pictures of one mouse ear per group. (**E**) Representative H&E stained sections of each treatment and control group. Magnification = 100×, scale bar = 250 µm. Immunohistochemical staining for neutrophils was performed with a primary monoclonal rat anti-mouse Ly-6G antibody. Magnification = 200×, scale bar = 100 µm.

This study aimed to establish an IL-17A and IL-36γ intradermal ear injection model of psoriasis to elucidate the role of both cytokines in the complex pathogenesis of the disease. While former studies used IL-23 to induce psoriasis in mouse models [36,37,38], we went a step further in the cytokine cascade by injecting IL-17A and IL-36γ. Injection of IL-17A or IL-36γ alone led to a significant increase of the ear thickness from day two onwards (Figure 1A,B). The increase was slightly larger in the IL-36γ group. Injection of both IL-17A + IL-36γ resulted in the largest increase in ear thickness. In the PBS control group, only a slight increase (about 10%) occurred due to the injection stimulus. Ear thickening was maximal by day four in all groups. In psoriasis, epidermal hyperplasia, known as acanthosis is a typical histological finding. To evaluate if these findings were also evident in our model, epidermal thickness was measured in two representative H&E-stained sections from each mouse. Epidermal thickening was strongest in mice treated with the combination of IL-17A + IL-36γ, followed by IL-36γ and IL-17A single treatments (Figure 1C). Thus, an additive effect after injection of the combination of IL-17A + IL36γ was observed in both ear thickness and epidermal thickness measurements.

While the increase in ear thickness is an indicator of vascular permeability and oedema, an increased epidermal thickness could be attributed to differences in the number of actively proliferating cells within the epidermal layers of the groups. The ear and epidermal thickness results were concordant with visually apparent skin changes (Figure 1D). The most substantial effects, such as desquamation and incrustations with erythema, were seen in mice that were treated by the combination of both cytokines. Similar changes were seen in the IL-36γ group. IL-17A was only able to induce minor changes concerning epidermal desquamation. To observe if we can induce psoriasis-like skin changes in the mouse ear after the injection of the indicated cytokines, ear sections were evaluated histologically and infiltrating immune cells were determined by immune-staining (Figure 1E). IL-17A + IL-36γ-treated mice showed typical signs of psoriasis, including hyperparakeratosis, as well as an acanthosis with spongiosis and dermal oedema. Immunohistochemical analysis with a Ly-6G antibody demonstrated massive recruitment of neutrophils. IL-36γ or IL-17A provoked more eczematous-like skin changes along with mild epidermal hyperplasia with intact stratum granulosum and spongiosis compared to the combined administration of the cytokines. We noted that IL-36γ alone led to the recruitment of neutrophils, whereas IL-17A-stimulated ears showed only immigration of a few neutrophils at the injection area, where the skin was injured.

**Figure 2:**
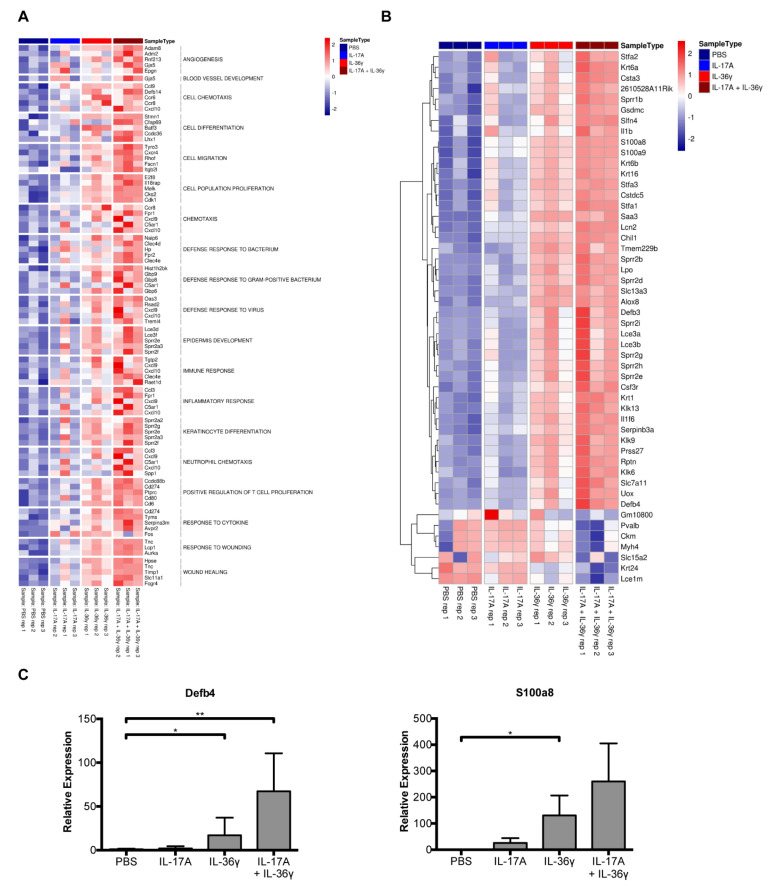
Identification of differentially expressed psoriasis-associated genes in mice treated with IL-17A and IL-36γ. (**A**) Heat map generated from RNAseq data reflecting gene expression values in four conditions (treatment with PBS, IL-17A, IL-36γ, or both cytokines). Nineteen gene sets relevant for psoriasis were selected. The top five significantly regulated genes (with a fold change greater or equal to two and a *p*-value less than 0.01) of every gene set are presented. The 12 columns of the heat map from left to right correspond to individual mice of the indicated treatment. (**B**) Heat map of the top 50 differentially expressed genes. The 12 columns of the heat map from left to right correspond, respectively to PBS, IL-17A, IL-36γ, IL-17A + IL-36γ. (**C**) The expression of the genes encoding S100a8 and Defb4 (ortholog of human hBD2) after treatment with IL-17A and IL-36γ as indicated was analyzed by RT-qPCR analyses. * *p* < 0.05, ** *p* < 0.01.

Next, we performed gene expression profiling using RNA-Seq. Nineteen gene sets that play a pivotal role in the psoriasis pathomechanism were selected (Figure 2A). While the IL-17A-treated mice showed only slight gene expression effects, mice injected with IL-17A + IL-36γ exhibited significant upregulations in all selected psoriasis-relevant gene sets. In addition, we displayed the top 50 differentially expressed genes that were found in the IL-17A + IL36γ group. Many of these genes, such as Defb4 and S100a8, are key players in the pathomechanism of psoriasis (Figure 2B). The upregulated expression of genes encoding antimicrobial peptides (AMPs) like S100a8 and Defb4 (ortholog of human hBD2) was additionally confirmed by an independent qRT-PCR analysis (Figure 2C). A more than additive effect was observed upon injection of both IL-17A and IL-36γ. These findings validate the RNA-seq analysis.

**Figure 3:**
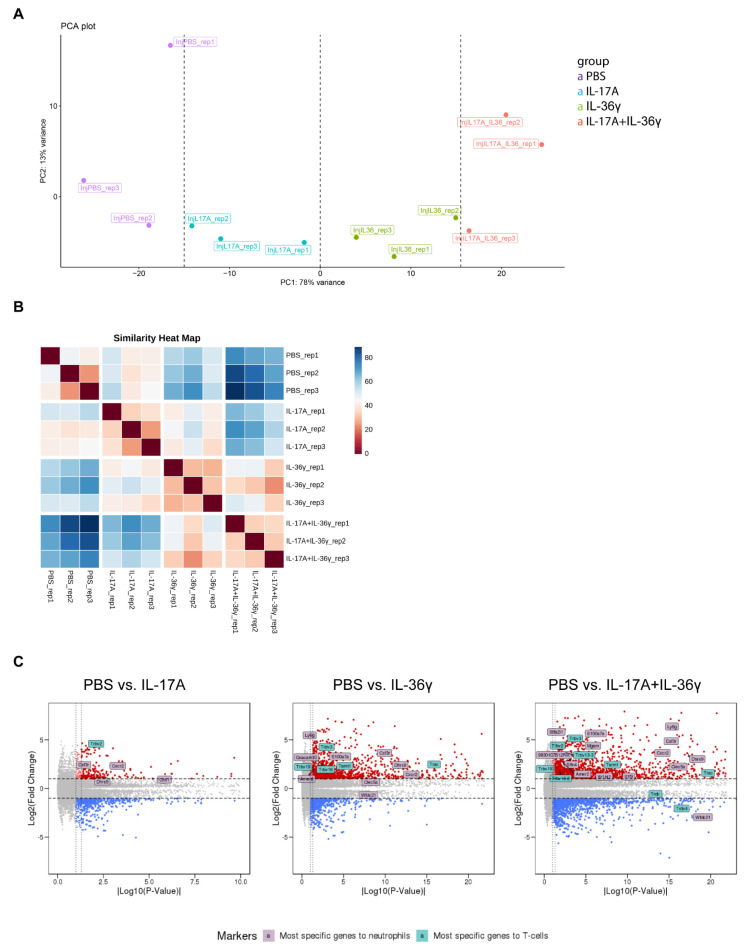
Principal components analysis (PCA), similarity heat map and gene expression analysis of immune cells. (**A**) PCA plot of all 12 samples using variance stabilizing transformation. (**B**) Similarity heatmap with dark red indicating identical or very similar, and dark blue very different gene expression changes. (**C**) Volcano plots of differentially expressed genes. Indicated are genes that are expressed selectively in T cells and neutrophils. Genes with increased and decreased expression (*p* < 0.05) are in red and blue, respectively.

We performed principal components analysis (PCA) on the expression profiles of the 12 samples, then we selected the first two principal components (describing ~90% of the variance in the data). The corresponding PCA plot showed that replicates of the same treatment group were generally clustering. All groups were well separated along PC1, contributing 78% to the data variance. We concluded that PC1 explains the effect of the treatment, with the effect increasing from left to right. In contrast, we supposed that differences along PC2 were mainly due to technical effects as well as natural variability (Figure 3A).

The similarities of the differentially treated groups were further confirmed by the Similarity Heat Map (Figure 3B). The replicates from the same treatment group showed high concordance.

As mentioned before, neutrophils and T-cells play a crucial role in developing and maintaining psoriasis inflammation. We expected that in the RNA-seq dataset, obtained from biopsies of mouse ears that include infiltrating cells, signatures of both neutrophils and T cells should be detectable. Therefore, genes that are preferentially expressed either in neutrophils or in T cells were evaluated [39,40]. After injection of IL-17A, only Trbv2 (specific for T cells) showed a statistically significant increase as visualized in the volcano plot (Figure 3C). Four neutrophil-specific genes, including Cxcr2, were upregulated. Mice treated with IL-36γ revealed significantly more genes with expression relatively specific to T cells and neutrophils that were upregulated. In mice treated with both cytokines, the highest number of genes with expression relatively specific to neutrophils and T cells was identified when compared to mice treated with IL-36γ or IL-17A alone, confirming the stainings shown in Figure 1E.

## 4. Discussion

The WHO classifies psoriasis as one of the most serious non-communicable diseases. It is characterized by aberrant and enhanced proliferation of epidermal keratinocytes driven by immune mediators including IL-23, IL-17 and IL-36 [1]. These cytokines are organized hierarchically, with IL-36 being the downstream effector [41]. Previous studies have used mouse models, in which the injection of IL-23 induced a psoriatic phenotype to investigate the molecular key mechanisms of the disease [36,37,38]. In this context, a former study elucidated a role for IL-36 signaling in the IL-23/TH17-signaling axis by inhibiting skin inflammation in an IL-23 ear injection model with an anti-mouse IL-36R monoclonal antibody [42]. Our present study aimed to establish a murine intradermal ear injection model to investigate the effects of IL-36γ and IL-17A in the pathomechanism of psoriasis.

We demonstrated that IL-36γ and IL-17A individually led to a significant increase in ear and epidermal thickness, which was further increased by the combined administration of both cytokines. This strong increase in ear thickness is also observed in IL-23-induced mouse models of psoriasis described in the literature, concordant with our results [34]. Injection of each cytokine alone provoked eczematous-like skin changes, whereas the combination leads to a psoriasis-like phenotype.

The role of neutrophils in psoriasis has been known for a long time. Neutrophil chemotaxis is significantly upregulated in psoriasis patients [43]. The infiltration of neutrophils and the formation of micro-abscesses is a hallmark feature of psoriasis. In our psoriasis model, immigration of neutrophils occurred after injection of both IL-17A + IL-36γ, but also in response to IL-36γ alone, albeit to a lesser extent. This confirms that IL-36 cytokines are important for the recruitment and activation of neutrophils [30]. It was also reflected at the gene expression level. We found that genes such as *Cxcl9* and *Cxcl10* were significantly upregulated, which are both important chemo-attractants for neutrophils. It has been reported that intraperitoneal treatment with a neutrophil-depleting antibody (anti-Ly-6G) caused a dramatic reduction in epidermal thickness in a psoriasis mouse model and suggested that blocking neutrophil function may have therapeutic benefit in inflammatory human skin disorders [44]. Therapies frequently used in psoriasis patients such as dimethylfumarate or apremilast can inhibit chemokines like Cxcl9 and Cxcl10, which highlights the role of these two chemokines in psoriasis [45,46].

The role of TH1 in the disease is well known. A feedback loop has been postulated in which TH1 activation leads to the release of IFNγ and TNFα, which in turn stimulate the secretion of Cxcl10 by keratinocytes, lymphocytes, and other immune cells [44]. The release of Cxcl10 promotes activation of T cells which perpetuates the immune cascade [47]. The recruitment of T cells in psoriasis is consistent with the observed changes in gene expression in our ear injection model as T cell-specific marker genes were up-regulated. T cells play the central role for the adaptive immune system and are crucial for maintaining inflammation in psoriasis patients.

Genes involved in angiogenesis and blood vessel development pathways, which are associated with psoriasis, are significantly upregulated by the combination of IL-17A + IL-36γ, and to a lesser extent in response to IL-36γ alone. Our results are in line with findings that highlighted the role of IL-36γ in vascular activation, and the induction of angiogenesis in a VEGF-A-dependent manner in the context of psoriatic lesions [48]. Additionally, IL-17A stimulates the expression of pro-angiogenic factors, including VEGF [49]. In our model, genes associated with angiogenesis and blood vessel development were only slightly upregulated by IL-17A.

The stimulation with IL-17A + IL-36γ upregulated numerous psoriasis-associated genes. This included, for example, the RIKEN cDNA 2610528A11 gene, which is a marker for psoriasis in humans. Moreover, the mRNA for 2610528A11RIK (Gpr15l) was significantly decreased upon treatment with the antibody AIN457 (Secukinumab), which was simultaneously accompanied by a marked improvement in clinical findings [50]. Furthermore, we detected an upregulation of *Il1b*, whose proinflammatory role in psoriasis has been described previously [51,52].

The upregulation of genes encoding proteins of the S100 family is in line with previous studies, which showed that this protein family plays a crucial role in psoriasis, especially in inflammation, angiogenesis, and keratinocyte proliferation [53]. S100A8/A9 often exists in a heterodimeric form. They are derived from neutrophils, macrophages, and keratinocytes and stimulate the recruitment of neutrophils [54]. Therefore, it is not surprising that in mice, in which S100 genes are upregulated (in response to IL-36γ as well as the combination of IL-17A + Il-36γ), significantly more neutrophils were detected in the histological sections.

Furthermore, a significant upregulation of genes belonging to the Krt family involved in keratinization, and keratinocyte differentiation was found. Comparable to our study, an upregulation of *Krt16* was demonstrated in a three-dimensional psoriasis mouse model [55]. However, this was after stimulation with TNFα and IFNγ. In our model, *Krt16* was upregulated in response to IL-36γ, either alone or in combination with IL-17A.

IL-17A and IL-36γ induced the expression of *Lce3a* and *Lce3b*, which are expressed only in the epidermis and in oral epithelia. The encoded proteins are not only relevant in the late cornified envelope, but also possess antimicrobial activity [56]. In lesional psoriasis skin, the expression of LCE3A, LCE3B and LCE3C is markedly increased, which is consistent with our findings [56].

*Defb3* and *Defb4* were upregulated by IL-36γ and synergistically by co-stimulation with IL-17A + IL-36γ. Interestingly no upregulation was detected after stimulation with IL-17A alone. Our results differ from a previous study [57], in which treatment of NHEKs with IL-17A induced the expression of proinflammatory mediators, such as DEFB4.

## 5. Conclusions

In conclusion, intradermal injection of IL-17A + IL-36γ into the ear pinnae of mice provides an in vivo model to investigate psoriasis, which mimics both histological and gene expression aspects of the disease. Our results strengthen the hypothesis that IL-17A and IL-36γ drive psoriatic inflammation via a synergistic interaction. In severe cases of psoriasis, the combined targeting of IL-36 and the IL-23–IL-17 axis may be a new beneficial therapeutic approach. Monoclonal IL-36 receptor-blocking antibodies such as Spesolimab have already proven their efficacy in generalized pustular psoriasis in Phase II clinical trials. Our established intradermal ear injection model can be utilized in the future to monitor the effects of various inhibitors, e.g., biologicals, and topically applied small molecules, that target the IL-17A and IL-36γ cytokine network.

## Data Availability

Bulk RNA-Seq data has been submitted to the Gene Expression Omnibus (GEO) repository (https://www.ncbi.nlm.nih.gov/geo/ (accessed on 28 May 2021)), accession no. GSE175732.
